# Plasma metabolites and physical function in patients undergoing hemodialysis

**DOI:** 10.1038/s41598-024-58522-9

**Published:** 2024-04-10

**Authors:** Ranjani N. Moorthi, Sharon M. Moe, Thomas O’Connell, Stephanie Dickinson, Sahir Kalim, Ravi Thadhani, Clary B. Clish, Tariq Shafi, Eugene P. Rhee, Keith G. Avin

**Affiliations:** 1 Indiana University School of Medicine, Indianapolis, IN USA; 2 Indiana University School of Medicine, Indianapolis, IN USA; 3 Department of Biostatistics, Indiana University, Bloomington, IN USA; 4 Department of Medicine, Massachusetts General Hospital, Boston, MA 02114 USA; 5 Broad Institute of MIT and Harvard, Cambridge, MA 02142 USA; 6 Department of Medicine, University of Mississippi Medical Center, Jackson, MI 39216 USA; 7 School of Health and Human Sciences, IUPUI, Indianapolis, IN USA

**Keywords:** Metabolites, Gait speed, Grip strength, Hemodialysis, Chronic kidney disease, Renal replacement therapy

## Abstract

Impaired physical function contributes to falls, fractures, and mortality among patients undergoing dialysis. Using a metabolomic approach, we identified metabolite alterations and effect size-based composite scores for constructs of impaired gait speed and grip strength. 108 participants incident to dialysis had targeted plasma metabolomics via liquid chromatography-mass spectrometry and physical function assessed (i.e., 4 m walk, handgrip strength). Physical function measures were categorized as above/ below median, with grip utilizing sex-based medians. To develop composite scores, metabolites were identified via Wilcoxon uncorrected *p* < 0.05 and effect size > 0.40. Receiver operating characteristic analyses tested whether scores differentiated between above/below function groups. Participants were 54% male, 77% Black and 53 ± 14 y with dialysis vintage of 101 ± 50 days. Median (IQR) grip strength was 35.5 (11.1) kg (males) and 20 (8.4) kg (females); median gait speed was 0.82 (0.34) m/s. Of 246 measured metabolites, composite scores were composed of 22 and 12 metabolites for grip strength and gait speed, respectively. Area under the curve for metabolite composite was 0.88 (gait) and 0.911 (grip). Composite scores of physical function performed better than clinical parameters alone in patients on dialysis. These results provide potential pathways for interventions and needed validation in an independent cohort.

## Introduction

Patients undergoing dialysis have poor skeletal muscle strength and slowed gait speed that increases the risk of functional dependence, frailty, fractures and falls^1–5^. In 183 patients new to dialysis, we have demonstrated low grip strength (median 27.0 ± 11.5 kg) and slow gait speed (median 0.78 m/s (0.64–0.94))^2^. In this cohort, multivariate analyses demonstrated low gait speed was associated with several clinical risk factors including overall health utility indices, diabetic nephropathy, and use of a walking aid. In another cohort of 277 patients undergoing dialysis, 41% of subjects showed a discrepancy between gait speed and grip strength, with low performance on one test and normal performance on the other test with a very weak correlation coefficient between these tests (R^2^ = 0.07)^6^. Further, the clinical risk factors for slow gait and low grip were distinct with diabetes and low serum albumin for slow gait speed and a history of cardiovascular disease and lower body-mass index for low grip strength. The discrepant findings between upper and lower extremity tests were further supported in a non-dialysis CKD cohort (n = 385)^7^. Thus, muscle strength and gait speed are measures that impact muscle health but may or may not reflect similar metabolic adaptations with disease.

Skeletal muscle contraction is predicated upon multiple cellular energy pathways that feed into oxidative phosphorylation and substrate level phosphorylation reactions that generate adenosine triphosphate (ATP). ATP production occurs from three energy sources, including carbohydrates, amino acids, and fatty acids. The downstream pathways from multiple energy systems are numerous, making identification of dysfunctional pathways difficult. However, a broad survey of the metabolome has the potential to distinguish these pathways, as metabolites represent the downstream expression of the genome, transcriptome, and proteome, and provide proximate insight to the disease risk/phenotype^8^. Prior studies have shown that metabolites associated with cellular energy are altered in patients undergoing dialysis and associated with adverse outcomes including mortality^9–12^. We used similar liquid chromatography-mass spectrometry (LC–MS) based metabolomics methods to identify plasma metabolites of relevance to physical function measures as an assessment of skeletal muscle health in those on dialysis.

Studies utilizing metabolomics to study muscle health, including skeletal muscle mass, strength or function performed in healthy older adults have shown alterations in amino acids (e.g., leucine, isoleucine, and glutamic acid)^13–15^ and lipids^15^. A study in young to middle aged women assessed muscle mass and function and found nine metabolites, mainly amino acids and lipids, associated with muscle mass, four of which were also associated with muscle strength^16^. In participants with type 2 diabetes mellitus, poor physical function was associated with higher levels of 3-methyl histidine, alanine, arginine, glutamic acid, ethanolamine, sarcosine, and tryptophan indicative of protein/amino acid perturbations^17^. However, to our knowledge, there is no prior report of utilizing metabolomics to discriminate between levels of muscle health/physical function in patients undergoing dialysis.

Physical function/health is complex and impacted by multiple physiological systems and thus a single gene, protein or metabolite is unlikely to explain the complexities, nor be an effective therapeutic target. Therefore, we sought to develop a panel of metabolic alterations that underlie impaired physical function, i.e., slow gait speed and/or weak grip strength, in patients with end-stage kidney disease (ESKD) new to dialysis. We hypothesized that metabolites will be different for those with low versus high grip strength and gait speed in a cohort of patients new to outpatient dialysis. We performed an exploratory study to identify a panel of metabolites that underlie physical function, that could inform future personalized exercise and nutraceutical interventional trials targeting musculoskeletal health in those with CKD.

## Results

### Demographics

Subjects overall had a mean age of 53.4 ± 13.9 years, 54% were male, 77.4% were Black, and mean dialysis vintage was 101.2 ± 49.7 days (Table 1). The median grip strength for males was 35.5 kg (range 10.5–55 kg) and females were 20 kg (range 12–39.5 kg). The median usual gait speed was 0.82 m/s (range 0.23–1.4 m/s). Study participants' characteristics are shown in Table 1.

**Table 1:** Participant characteristics base upon median gait speed or grip strength (N = 108).

Subject characteristics	All	Gait speed < 0.82 m/s	Gait speed ≥ 0.82 m/s	*p*	Below sex-specific median grip strength	At or Above sex-specific median grip strength	*p*
Age, mean ± SD	53 ± 14	58 ± 13	49 ± 13	**0.002**	59 ± 13	49 ± 13	**< 0.001**
Male, n (%)	54 (50%)	19 (35%)	35 (65%)	**0.004**	27 (55%)	27 (46%)	0.44
Race, n (%)				1.000			0.27
Black	82 (77%)	41 (50%)	41 (50%)		34 (41%)	48 (59%)	
White	22 (21%)	11 (50%)	11 (50%)		13 (59%)	9 (41%)	
Other	2 (2%)	1 (1%)	1 (1%)		1 (50%)	1 (50%)	
Ethnicity, Hispanic, n (%)	8 (7%)	3 (38%)	5 (63%)	0.72	3 (38%)	5 (63%)	0.73
BMI, mean ± SD	28 ± 7	28 ± 7	28 ± 6	0.99	26 ± 6	29 ± 7	**0.049**
Residual Renal Function/ Producing Urine, Yes/No/Not Listed	90/5/13	49/1/4	40/4/10	0.812	40/5/3	42/9/9	0.365
Days since first outpatient dialysis treatment, mean ± SD	101 ± 50	101 ± 52	101 ± 48	0.97	97 ± 48	105 ± 51	0.42
Dialysis Access Permanent/Cath/Not Listed	40/56/12	22/30/2	18/26/10	0.889	19/27/2	23/27/10	0.643
Dialysis Dose_Kt/v	1.59 ± 0.40	1.66 ± 0.43	1.53 ± 0.36	0.202	3.60 ± 0.57	3.71 ± 0.49	0.366
Albumin	3.67 ± 0.53	3.62 ± 0.57	3.70 ± 0.50	0.49	1.61 ± 0.34	1.58 ± 0.36	0.767
Diabetic nephropathy as ESRD cause, n (%)	36 (33%)	24 (66%)	12 (33%)	**0.024**	19 (53%)	17 (47%)	0.31
Overall Health Utility Index, mean ± SD	0.8 ± 0.2	0.7 ± 0.2	0.8 ± 0.2	**0.023**	0.8 ± 0.2	0.8 ± 0.2	0.13
Walking aid, n (%)	16 (15%)	16 (100%)	0 (0%)	** < 0.0001**	6 (38%)	10 (63%)	0.71
Diabetes mellitus, n (%)	47 (44%)	27 (57%)	20 (43%)	0.24	24 (51%)	23 (49%)	0.34
Cardiovascular disease, n (%)	53 (49%)	29 (55%)	24 (45%)	0.34	31 (58%)	22 (42%)	**0.01**
Hypertension, n (%)	97 (90%)	47 (48%)	50 (52%)	0.53	44 (45%)	53 (55%)	1.000
History of Cerebrovascular Disease, n (%)	15 (14%)	11 (73%)	4 (27%)	0.09	10 (66%)	5 (33%)	0.09
Peripheral vascular disease, n (%)	17 (16%)	10 (59%)	7 (41%)	0.60	8 (47%)	9 (53%)	1.000
Smoker, current or past, n (%)	48 (44%)	28 (58%)	20 (42%)	0.24	27 (56%)	21 (44%)	0.053

Values significant at *p* < 0.05 values are in bold.

### Grip strength metabolites

**Figure 1:**
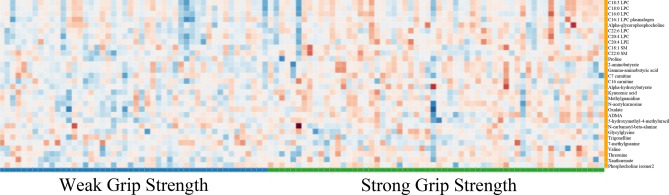
Grip Strength Heatmap: 30 significant metabolites visually depicted lower metabolite expression in those with low grip strength (Blue) as compared to those with higher grip strength (Green). The significant metabolites consisted of lipid and lipid-like molecules (LPC 22:6, LPC 20:4, LPC plasmalogen 16:1, LPE 20:4, SM 22:0, carnitine 16:0, SM 18:1, LPC 18:3, carnitine 7:0, LPC 18:0, LPC 16:0, alphaglycerophosphocholine), organic acids and derivatives (asymmetric dimethylarginine, gamma aminobutyric acid, oxalate, alpha-hydroxybutyrate/beta-hydroxybutyrate/ hydroxyisobutyrate, n-acetylcarnosine), organoheterocyclic compounds (kynurenic acid, xanthurenate, 7-methylguanine, 5-hydroxymethyl-4-methyluricil), alkaloids and derivatives (trigonelline), organic nitrogen compounds (methylguanidine), carboximidic acids (N carbomoyl beta alanine), amino acids and peptides (proline, valine, threonine and glycylglycine) and phosphocholine isomer 2.

Wilcoxon comparisons identified 30 of the 246 metabolites with *p* value < 0.05 comparing subjects above and below median grip strength (Table 2), with effect sizes ranging from 0.206–0.563. The metabolites represented different chemical classes including phospholipids, acylcarnitines, organic acids and amino acids. Heatmaps provide a visual depiction of the qualitative differences of the 30 selected metabolites, where those with weak grip strength had increased long-chain sphingomyelins and glycerophospholipids (Fig. 1). The composite score is the average across the scaled and transformed data for metabolites with a Wilcoxon *p* value < 0.05 and ES > 0.40; 22 metabolites achieved this criterion. These 22 metabolites resulted in a composite score ES of 1.19 (*p* < 0.001) comparing high/low grip strength. To demonstrate that the grip metabolites were unique, we also calculated a composite score using only the gait metabolites for the grip data, which resulted in a lower ES = 0.48 (*p* = 0.015).

**Table 2:** Key Metabolites between weak (low) and strong (high) median grip strength.

Metabolites (alphabetical order)	Wilcoxon *p* value	Cohen’s d Effect Size	Scaled & Transformed Data (Median)	
			Weak Grip	Strong Grip
2-aminobutyrate	0.049	0.295	11,389,082	14,908,415
5-hydroxymethyl-4-methyluracil	0.03	0.381	2111	2537
**7-methylguanine**	**0.015**	**-0.542**	**82,546**	**69,896**
ADMA	**0.044**	**0.392**	**99,657,503**	**113,624,013**
**Alpha-glycerophosphocholine**	**0.01**	**0.478**	**88,553**	**102,301**
**Alpha-hydroxybutyrate**	**0.008**	**0.563**	**139,871**	**171,554**
*C7 carnitine	0.031	0.33	5472	7384
**C16 carnitine**	**0.021**	**0.483**	**60,653**	**76,773**
C16:0 LPC	0.032	0.382	525,421,727	581,920,489
**C16:1 LPC plasmalogen**	**0.025**	**0.501**	**40,466**	**50,741**
**C18:0 LPC**	**0.04**	**0.442**	**2,689,328**	**3,014,587**
**C18:1 SM**	**0.014**	**0.486**	**944,913**	**791,938**
**C18:3 LPC**	**0.029**	**0.432**	**993,828**	**1,082,102**
**C20:4 LPC**	**0.005**	**0.561**	**980,097**	**1,252,246**
**C20:4 LPE**	**0.026**	**0.427**	**313,820**	**363,894**
**C22:0 SM**	**0.042**	**0.427**	**835,303**	**944,913**
**C22:6 LPC**	**0.014**	**0.478**	**80,524**	**100,988**
**Gamma-aminobutyric acid**	**0.011**	**0.439**	**2375**	**3297**
**Glycylglycine**	**0.043**	**0.431**	**167**	**245**
**Kynurenic acid**	**0.027**	**0.427**	**103,549**	**1,471,901**
**Methylguanidine**	**0.02**	**0.417**	**24,676**	**38,671**
*N-acetylcarnosine	**0.007**	**0.455**	**74,699**	**102,293**
N-carbamoyl-beta-alanine	0.012	-0.342	5300	4123
Oxalate	0.045	0.351	3,217,149	3,971,095
*Phosphocholine isomer2	**0.019**	**0.501**	**454**	**562**
**Proline**	**0.013**	**0.46**	**2,132,185**	**2,593,390**
**Threonine**	**0.025**	**0.414**	**30,472**	**35,086**
**Trigonelline**	**0.006**	**-0.523**	**1,522,587**	**845,979**
*Valine	0.044	0.206	106,310,445	122,883,378
**Xanthurenate**	**0.002**	**0.464**	**61,135**	**119,730**

*Indicates metabolites consistent for both gait speed and grip strength.

Bolded items indicate metabolites with ES > 0.4 that were included in the composite score.

### Gait speed metabolites

**Figure 2:**
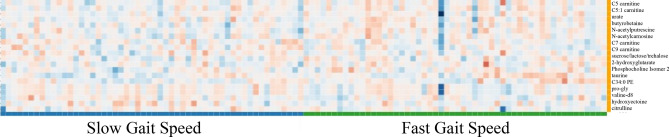
Gait Speed Heatmap. 20 significant metabolites visually resulted in a less striking difference in metabolite concentrations when comparing slow gait speed (Blue) to fast gait speed (Green). The 20 significant metabolites consisted of acylcarnitines (C5, C5:1, C7, C9, PE 34:0), organic acids and derivatives (prolyl-glycine, ornithine, taurine, N-acetylputrescine, N-acetylcarnosine, creatine, 2-hydroxyglutarate, citrulline, valine), organic nitrogen compounds (trimethylamine-n-oxide), benzenoids (hydroxyectoine), nucleic acid (urate), carbohydrate (sucrose), lipid and lipid-like molecules (Butyrobetaine) (and phosphocholine. We have included the heatmap of the selected 20 metabolites to provide a visual depiction of the qualitative differences; there was a lack of stark differences depicted between those with slow versus fast gait speed.

Wilcoxon comparisons identified 20 of the 246 metabolites with *p* value < 0.05 when grouped by above and below median gait speed (Table 3) with ES ranging from 0.205–0.559. Broadly, these metabolites consisted of acylcarnitines (C5, C5:1, C7, C9, PE 34:0), organic acids and derivatives (prolyl-glycine, ornithine, taurine, N-acetylputrescine, N-acetylcarnosine, creatine, 2-hydroxyglutarate, citrulline, valine), organic nitrogen compounds (trimethylamine-n-oxide), benzenoids (hydroxyectoine), a nucleic acid (urate), a carbohydrate (sucrose), lipid and lipid-like molecules (Butyrobetaine), and phosphocholine. The heatmap of the selected 20 metabolites depicts a lack of stark differences depicted between those with slow versus fast gait speed (Fig. 2). The composite score, which is the average of the normalized data across the metabolites with a *p* value < 0.05 and ES > 0.40, included 12 metabolites that achieved this criterion. These 12 metabolites resulted in a composite score with ES of 1.38 (*p* < 0.001) comparing fast/slow gait speed. To demonstrate that the gait metabolites were unique, we calculated a composite using grip metabolites for the gait data, which resulted in a lower ES = 0.54 (*p* = 0.006).

**Table 3:** Key metabolites between fast and slow median gait speed.

Metabolites	Wilcoxon *p* value	Cohen’s d Effect Size	Slow Gait	Fast Gait
			Scaled and Transformed Data (Median)	
**2-hydroxyglutarate**	**0.035**	**-0.403**	**193,887**	**136,763**
Butyrobetaine	0.034	0.295	605,460	703,598
**C34:0 PE**	**0.049**	**0.419**	**62,615**	**73,313**
C5 carnitine	0.032	0.376	114,268	138,701
C5:1 carnitine	0.034	0.25	27,574	34,970
*C7 carnitine	**0.002**	**0.559**	**5778**	**7613**
**C9 carnitine**	**0.019**	**0.439**	**19,826**	**28,006**
**Citrulline**	**0.041**	**-0.438**	**254,442**	**218,413**
Creatine	0.03	-0.391	573,863	392,584
**Hydroxyectoine**	**0.037**	**-0.42**	**69,687**	**61,071**
*N-acetylcarnosine	**0.028**	**0.427**	**83,192**	**104,972**
N-acetylputrescine	0.026	-0.399	18,557	16,524
**Ornithine**	**0.015**	**-0.462**	**207,970**	**148,199**
*Phosphocholine isomer2	0.048	0.365	435	578
**Prolylglycine**	**0.014**	**0.548**	**2465**	**2786**
**Sucrose/lactose/trehalose**	**0.018**	**0.474**	**3,661,592**	**2,113,983**
**Taurine**	**0.022**	**0.501**	**387,731**	**441,559**
Trimethylamine-N-oxide	0.036	-0.343	973,003	786,738
Urate	0.046	0.205	1,565,581	1,699,259
*Valine	**0.045**	**0.448**	**108,818,305**	**113,654,326**

*Indicates metabolites consistent for both gait speed and grip strength.

Bolded items indicate metabolites with ES > 0.4 that were included in the composite score.

### Metabolite profile performed better than clinical characteristics as predictors of grip strength and gait speed

ROC analyses for the prediction of grip strength and gait speed were developed for clinical variables alone, metabolites alone, and clinical variables combined with metabolites. Grip strength clinical variables of age, BMI, and history of cardiovascular disease resulted in an AUC of 0.749, while only grip metabolites had an AUC = 0.911, and combined clinical variables with grip metabolites had an AUC = 0.937 for prediction of grip strength. For gait speed prediction, clinical variables alone (sex, age, cause of kidney disease and healthy utility index score) resulted in an AUC of 0.778, whereas the gait metabolites alone had an AUC = 0.880 and the combined clinical variables with gait metabolites had an AUC = 0.891. The AUCs for both grip strength and gait speed were greater for only metabolites compared to clinical variables and were similar to the combined clinical variables with metabolites AUCs.

## Discussion

This study represents the first comprehensive untargeted evaluation of the association of metabolite profiles with physical function in patients on dialysis. Untargeted metabolomic approaches have been performed in multiple studies in those who are non-CKD older adults but have not been explored in those with CKD or new to dialysis^18,19^. In this study, we identified 20 metabolites for gait speed and 30 metabolites for grip strength with either a *p* < 0.05 or ES > 0.4. Only four metabolites were consistent between gait speed and grip strength (C7 carnitine, valine, phosphocholine isomer 2, n-acetylputrescine), but none of these metabolites achieved significance for both *p* value and ES. The nominal number of overlapping metabolites reinforces the notion that gait speed and grip strength are two different constructs. Composite scores were constructed by combining the multiple metabolites that achieved both *p* value and ES criteria (22 for grip strength and 12 for gait speed) to reflect physical function in patients who are incident to dialysis. The composite scores for the metabolites had diagnostic abilities per AUC scores of 0.91 and 0.88 respectively for grip strength and gait speed, better than clinical-demographic predictors alone and minimally different from combining clinical variables with the metabolites. Although the AUCs were promising, further work is required to validate the metabolite panel in a larger cohort. Collectively, these panels offer insight into the pathogenesis and potential target metabolites for improving physical function in those incident to dialysis, and if prospectively validated, diagnostic potential.

The intent of this pilot study was to determine if there were metabolites associated with physical function as captured by gait speed and grip strength. Gait speed is a multi-system measure that reflects muscle strength, bone-muscle interaction, neuromuscular control, and balance. The measure is considered the 5th vital sign in geriatrics, and in CKD is associated with morbidity and mortality^1,5,20^. In contrast, grip strength is an isolated measure of muscle function, also associated with all-cause mortality in patients undergoing hemodialysis^21–23^. We anticipated metabolites would be unique to each physical function construct because of the contrast in task requirements between gait speed and grip strength. Muscle contraction, required for both constructs, requires energy with three major sources: fatty acid oxidation, glycolysis, and protein metabolism and thus we focused on these pathways, although there were no metabolites identified in the glycolytic pathway.

Fatty acid oxidation metabolites were identified for both gait speed and grip strength. Gait speed identified four acylcarnitines (i.e., C7, C9, C5, C5:1), with ES ranging from 0.25 to 0.56 (positively associated with faster gait speeds), while grip strength identified two medium-long chain acylcarnitines (C16, C7), and nine medium-long chain lipid derivatives (i.e., C20:4 LPC, C22:6 LPC, C18:1 SM, C16:1 LPC plasmalogen, C20:4 LPE, C18:3 LPC, C16:0 LPC, C18:0 LPC, C22:0 SM) with ES ranging from 0.33 to 0.56 (positively associated with stronger gait strength). Fatty acid oxidation metabolites have been studied in the context of physical function in non-CKD populations, but there is a lack of consistent directions. In a study of 77 older men with a mean age of 79 years and average BMI of 28.4 kg/m^2^, higher acylcarnitine scores were associated with lower gait speeds^19^. In disease conditions such as heart failure, higher acylcarnitines were associated with a more pronounced disease state^24,25^. In a study with 43 community-dwelling older adults and age- and sex- matched controls higher plasma concentrations of medium- and long-chain acylcarnitines were associated with higher risk of lower extremity functional impairment by the short physical performance battery (SPPB) test that includes gait speed, but gait speed was not individually provided^26^. In this study, elevated acylcarnitines levels were positively associated with both gait speed and grip strength, which appears to be a unique feature in patients with CKD.

Grip strength was positively associated with taurine and valine, suggestive of altered protein metabolism which has been previously established in CKD^27^. Taurine is an amino acid with a moderate ES of 0.50, that defends against lipid induced oxidative stress. Taurine supplementation decreased lipid peroxidation marker malondialdehyde in rats with diabetes^28,29^. Treadmill running increased lipid peroxidation in rats but was mitigated by oral taurine supplementation^30^. The impact taurine may have on physical function draws from non-CKD preclinical and clinical studies, but is not yet noted in the CKD population and warrants further investigation. Additional metabolites that were associated with stronger grip strength were xanthurenate, kynurenic acid, and methylguanidine. Kynurenic acid is considered a uremic toxin, so the association with higher strength was surprising^31^. However, a study that isolated skeletal muscle mitochondria from healthy mice found that exposure to varying doses of L‐kynurenine, kynurenic acid, and methylguanidine decreased mitochondrial OXPHOS with no effect upon pyruvate dehydrogenase activity. Kynurenine metabolism utilizes a PGC-1α1-dependent mechanism to improve glucose oxidation and may have a beneficial effect upon skeletal muscle despite categorization as a uremic toxin^32^. Methylguanidine is exogenously provided through meat intake and endogenously by conversion from creatinine and arginine and has been suggested to be a uremic toxin^33^. Methylguanidine is the end-product of the reaction from guanidinoacetic acid to creatine to creatinine. Increased skeletal muscle utilization may place greater demand upon creatine and subsequent downstream accumulation of methylguanidine. Although this information supports the notion that metabolites along the kynurenine- tryptophan pathway could improve muscle strength, further studies are needed on the impact of uremic toxins on muscle metabolism and function.

Our study has several strengths and limitations. Strengths of this study are that both metabolite and physical function measures were concurrently collected from > 100 patients, distributed across sex and representative of an inner-city dialysis population. With the median number of days since the start of dialysis at 100 ± 46 days, we can exclude some of the variability in metabolites and muscle health with prolonged dialysis vintage. Another strength is use of a robust LC–MS platform for metabolite measures utilized extensively in kidney disease research^34,35^. The measurement of > 200 metabolites across all key cellular energy pathways provides a comprehensive overview in this discovery study. Limitations include being a cross-sectional analysis, use of plasma rather than muscle tissue, not accounting for inflammatory status, exclusion of drug metabolites, and a lack of healthy controls. Blood was collected pre-dialysis without controlling for diet or fasted/fed state at a single timepoint. We also acknowledge that selecting a set of metabolites out of 200 candidates based on unadjusted *p* < 0.05 and ES > 0.4 will identify up to 5% false positives in this sample. The scientific implementation and interpretation should be performed with careful consideration given the exploratory nature of the study and the potential influence of unaccounted confounding factors. A future validation study should identify additional confounding factors that may also contribute to differences in metabolic profiles and physical function. An additional limitation is the lack of a validation cohort which will be required prior to implementation of interventional studies to augment physical function.

In this study, we developed a composite score that represents physical function with metabolites unique for each measure of gait speed and grip strength in patients who are incident to dialysis. We identified 22 metabolites for grip strength and 12 metabolites for gait speed with composite score ES of 1.19 and 1.38, respectively. These were very large ES unique to each physical measurement indicating no overlap of specific metabolites, although alterations in fatty acid oxidation and protein synthesis were observed in both measures. Although some of the identified metabolites are known uremic toxins, many are novel and the role of these metabolites in muscle health is yet to be clearly elucidated. Further validation studies are warranted surrounding the selection of metabolites identified. Given the complexity of the systems involved in physical function, the use of panels of metabolites associated with physical function offers a fresh opportunity to offer insight into pathophysiology for those with CKD. A future direction of this work is to utilize these metabolic profiles to predict change over time (response/non-response) from an exercise or other interventional strategies (i.e., nutraceuticals) intended to improve physical functioning. Novel approaches such as this are vital to address both the physical and economic burden of impaired mobility in those with CKD.

## Methods

### Study design

Subjects who were enrolled in the Indiana-University Longitudinal Study of Incident Dialysis (IU-LUCID) were recruited from outpatient dialysis units affiliated with Indiana University Health Nephrology and located in inner-city areas^2^. Briefly, eligible participants were > 18 years old, started dialysis within the past 6 months, and had both plasma metabolites and physical function measurements collected at study initiation. The IU-LUCID study was approved by the Institutional Review Board at Indiana University and all procedures were performed in accordance with appropriate Guidelines and regulations and all participants were provided written informed consent. Clinical and demographic variables of sex, race, and smoking status were self-reported. Body mass index (BMI) was calculated based on in-person measurements of height (meters) and weight (kilograms). The ESKD cause was categorized into “diabetic nephropathy”, or “other cause” based on subjective interview and chart review. Co-morbidities such as diabetes, peripheral vascular disease, cardiovascular disease, cerebrovascular disease, and hypertension were obtained by self-report and chart review. The full cohort was previously described that only included those on hemodialysis^2^, only those with collected physical function measures were used for the current analyses. The battery of physical function measures were collected on the same day following a blood draw. Blood tubes clotted over 60 min followed by centrifugation at 1200×*g* for 10 min, samples were then aliquoted and stored at − 80 °C.

### Muscle-related measures and health utility assessment

Our goal was to identify those most at risk for poor muscle function in an already compromised cohort. We utilized clinically relevant rationale for gait speed and grip strength groups. Gait speed and grip strength measures were performed before dialysis (i.e., afternoon, evening shift) and post-dialysis (early AM shift). Gait speed of 0.8 m/s is common marker for identifying a more vulnerable population with higher hospitalizations and greater dependence^36^; this coincided with our gait speed median 0.82 m/s. Grip strength medians as compared to a large healthy cohort were at the 20^th^ percentile for males and 16^th^ percentile for females, thus indicating lower physical function/health. Grip strength was measured using Jamar hand dynamometers (Lafayette Instrument Company, USA) with the Southampton Grip-Strength Measurement Protocol^37^. Briefly, subjects were seated upright with the elbow unsupported at 90º elbow flexion in grip position 2. Maximal grip was assessed by alternating hands between each trial, for a total of 3 trials; the maximum value of all trials was recorded. Grip strength has been shown to provide an estimate of overall muscle strength^38^.

### Gait speed

Gait speed was calculated by dividing the time (seconds) used to walk a 4-m distance. Subjects were instructed to walk with/without the use of an assistive device at their usual walking speed from the start to finish of the 4 m. Walking speed was performed twice, with the highest value used in this analysis^39^.

### Metabolite measures

Metabolomics analyses utilized two LC–MS methods to profile metabolites in plasma. For MS analyses in the positive ion mode, precipitation of 10 µL of plasma was carried out by adding nine volumes of 74.9:24.9:0.2 v/v/v acetonitrile/methanol/formic acid containing stable isotope-labeled internal standards (valine-d8, Isotec; and phenylalanine-d8, Cambridge Isotope Laboratories). Following centrifugation (10 min, 9,000 × g, 4 °C), 2 µL of supernatants were injected onto a 150 × 2 mm Atlantis HILIC column (Waters). The column was eluted isocratically at a flow rate of 250 µL/min with 5% mobile phase A (10 mM ammonium formate and 0.1% formic acid in water) for 1 min, and after that with a linear gradient to 40% mobile phase B (acetonitrile with 0.1% formic acid) over 10 min. Electrospray ionization using full scan analysis over m/z 70–800 on a Q Exactive/Exactive Plus orbitrap mass spectrometer (Thermo Fisher Scientific) was used for MS analyses. For MS analyses in the negative ion mode, 30 µL of plasma was prepared via protein precipitation with the addition of four volumes of 80% methanol containing inosine-15N4, thymine-d4 and glycocholate-d4 internal standards (Cambridge Isotope Laboratories); 10 µL of centrifuged (10 min, 9000×*g*, 4 °C) supernatants were injected onto a 150 × 2.0 mm Luna NH2 column (Phenomenex). The column was eluted at a flow rate of 400 µL/min with initial conditions of 10% mobile phase A (20 mM ammonium acetate and 20 mM ammonium hydroxide in water) and 90% mobile phase B (10 mM ammonium hydroxide in 75:25 v/v acetonitrile/methanol). This was followed by a 10-min linear gradient to 100% mobile phase A. MS analyses were carried out using electrospray ionization in the negative ion mode using full scan analysis over m/z 60–750 on a Q Exactive/Exactive Plus orbitrap mass spectrometer (Thermo Fisher Scientific). Untargeted metabolomics identified 276 metabolites, with 30 metabolites being excluded due to a lack of physiologic relevance e.g., drug related metabolites or if they were missing in > 20% of subjects with metabolite data. Therefore, 246 metabolites were included in the final analysis.

### Statistical analysis

Subject characteristics were described using mean, standard deviation (SD), median, and frequencies as appropriate. Descriptive analyses were used to compare subject characteristics between above or below the median, with χ^2^ test and t-tests using SPSS. Grip strength was defined as above the group median (i.e., strong) and below (i.e., weak) based upon sex-dependent classifications. Gait speed was defined as above the group median (i.e., fast) and below (i.e., slow). We utilized a freely accessible software package, visualization and integration of metabolomics experiments (Viime)^40^, to log2 transform and Pareto scale the data for statistical comparisons and composite score development. We compared mean differences between the two groups using Wilcoxon tests. Cohen’s *d* effect sizes (ES) were calculated for each of the identified metabolites and corrected to maintain a positive connotation (i.e., negative effect ES was multiplied by − 1)^41^. A negative connotation indicated that the metabolite was higher in the slower or weaker groups as compared to the faster or stronger groups; a positive connotation was simply the reverse conditions. Metabolites were selected for the composite by achieving the following: (1) Wilcoxon test *p* < 0.05 and (2) ES > 0.40 which is classified as a “medium” effect size^42^. *p* values for Wilcoxon tests are unadjusted for multiple comparison; 0.05 is used as a convenient cut-off criterion for selecting candidate metabolites and not a formal conclusion of significance testing. As stated by Saville, when considering multiple comparison issues there is consistent standard or only one answer, but rather the process utilized should be identified by each investigator^43^. We are reporting the exact, uncorrected *p* values (rather than, e.g., “*p* < 0.05” or “NS”) enables readers to make their own judgments about statistical significance. This analysis and reporting are consistent with published guidelines for statistical reporting^44^. Recognizing that some random metabolites are possible, these metabolites are initial prospects needing future validation.

To calculate the cumulative score for both grip strength and gait speed the transformed and scaled data for each of the selected metabolites were averaged (i.e., metabolites with ES greater 0.4 and *p* < 0.05). The average of the scaled data was then calculated using the same Cohen’s d calculation, and is referred to as a “composite score”. Additionally, machine learning methods (logistic regression for classification) developed receiver operating characteristic (ROC) curves to test the ability of the metabolite panel to discriminate between the levels of gait speed or grip strength in cross-sectional analyses. To further demonstrate the importance of the metabolite profiles, we compared the areas under the curve (AUC) between the metabolite profile and subject characteristics that were statistically significant at a *p* < 0.05 in the t-test comparisons for both grip strength and gait speed groups.

## Data Availability

The datasets used and/or analyzed during the current study are available from the corresponding author upon a reasonable request.

## References

[1] Kuki A (2019). Association of gait speed and grip strength with risk of cardiovascular events in patients on haemodialysis: A prospective study. BMC Nephrol..

[2] Moorthi RN (2020). Mobility impairment in patients new to dialysis. Am. J. Nephrol..

[3] Kutner NG, Zhang R, Huang Y, Wasse H (2014). Gait speed and hospitalization among ambulatory hemodialysis patients: USRDS special study data. World J. Nephrol..

[4] Jamal SA, Leiter RE, Jassal V, Hamilton CJ, Bauer DC (2006). Impaired muscle strength is associated with fractures in hemodialysis patients. Osteoporos Int..

[5] Kutner NG, Zhang R, Huang Y, Painter P (2015). Gait speed and mortality, hospitalization, and functional status change among hemodialysis patients: A US renal data system special study. Am. J. Kidney Dis..

[6] Lee YH (2020). Gait speed and handgrip strength as predictors of all-cause mortality and cardiovascular events in hemodialysis patients. BMC Nephrol..

[7] Roshanravan B (2013). Association between physical performance and all-cause mortality in CKD. J. Am. Soc. Nephrol..

[8] Johnson CH, Ivanisevic J, Siuzdak G (2016). Metabolomics: Beyond biomarkers and towards mechanisms. Nat. Rev. Mol. Cell Biol..

[9] Kalim S (2018). Extended duration nocturnal hemodialysis and changes in plasma metabolite profiles. Clin. J. Am. Soc. Nephrol..

[10] Kalim S (2013). A plasma long-chain acylcarnitine predicts cardiovascular mortality in incident dialysis patients. J. Am. Heart Assoc..

[11] Titan SM (2019). Metabolomics biomarkers and the risk of overall mortality and ESRD in CKD: Results from the Progredir Cohort. PLoS ONE.

[12] Hu JR (2019). Serum metabolites and cardiac death in patients on hemodialysis. Clin. J. Am. Soc. Nephrol..

[13] Lustgarten MS, Price LL, Chale A, Phillips EM, Fielding RA (2014). Branched chain amino acids are associated with muscle mass in functionally limited older adults. J. Gerontol. A Biol. Sci. Med. Sci..

[14] Korostishevsky M (2016). Genomics and metabolomics of muscular mass in a community-based sample of UK females. Eur. J. Hum. Genet..

[15] Moaddel R (2016). Plasma biomarkers of poor muscle quality in older men and women from the Baltimore longitudinal study of aging. J. Gerontol. A Biol. Sci. Med. Sci..

[16] Zhao Q (2018). A joint analysis of metabolomic profiles associated with muscle mass and strength in Caucasian women. Aging (Albany NY).

[17] Calvani R (2020). Identification of a circulating amino acid signature in frail older persons with type 2 diabetes mellitus: Results from the metabofrail study. Nutrients.

[18] Gonzalez-Freire M (2019). Targeted metabolomics shows low plasma lysophosphatidylcholine 18:2 predicts greater decline of gait speed in older adults: The baltimore longitudinal study of aging. J. Gerontol. A Biol. Sci. Med. Sci..

[19] Lum H (2011). Plasma acylcarnitines are associated with physical performance in elderly men. J. Gerontol. A Biol. Sci. Med. Sci..

[20] Roshanravan B (2012). A prospective study of frailty in nephrology-referred patients with CKD. Am. J. Kidney Dis..

[21] Hwang SH, Lee DH, Min J, Jeon JY (2019). Handgrip strength as a predictor of all-cause mortality in patients with chronic kidney disease undergoing dialysis: A meta-analysis of prospective cohort studies. J. Ren. Nutr..

[22] Leal VO (2011). Handgrip strength and its dialysis determinants in hemodialysis patients. Nutrition.

[23] Vogt BP, Borges MCC, Goes CR, Caramori JCT (2016). Handgrip strength is an independent predictor of all-cause mortality in maintenance dialysis patients. Clin Nutr.

[24] Ahmad T (2016). Prognostic implications of long-chain acylcarnitines in heart failure and reversibility with mechanical circulatory support. J. Am. Coll. Cardiol..

[25] Hunter WG (2016). Metabolomic profiling identifies novel circulating biomarkers of mitochondrial dysfunction differentially elevated in heart failure with preserved versus reduced ejection fraction: Evidence for shared metabolic impairments in clinical heart failure. J. Am. Heart Assoc..

[26] Caballero FF (2021). Plasma acylcarnitines and risk of lower-extremity functional impairment in older adults: A nested case-control study. Sci. Rep..

[27] Workeneh BT (2006). Development of a diagnostic method for detecting increased muscle protein degradation in patients with catabolic conditions. J. Am. Soc. Nephrol..

[28] You JS, Chang KJ (1998). Effects of taurine supplementation on lipid peroxidation, blood glucose and blood lipid metabolism in streptozotocin-induced diabetic rats. Adv. Exp. Med. Biol..

[29] Ito T, Schaffer SW, Azuma J (2012). The potential usefulness of taurine on diabetes mellitus and its complications. Amino Acids.

[30] Dawson R, Biasetti M, Messina S, Dominy J (2002). The cytoprotective role of taurine in exercise-induced muscle injury. Amino Acids.

[31] Thome T (2019). Uremic metabolites impair skeletal muscle mitochondrial energetics through disruption of the electron transport system and matrix dehydrogenase activity. Am. J. Physiol. Cell Physiol..

[32] Agudelo LZ (2018). Kynurenic acid and gpr35 regulate adipose tissue energy homeostasis and inflammation. Cell Metab..

[33] Noda Y, Mankura M (2009). Inhibitory effect of antioxidants on hydroxyl radical generation from methylguanidine: An ESR study. Neurochem Res..

[34] Kalim S, Rhee EP (2017). An overview of renal metabolomics. Kidney Int..

[35] Kalim S (2015). Cross-sectional examination of metabolites and metabolic phenotypes in uremia. BMC Nephrol..

[36] Cruz-Jentoft AJ (2010). Sarcopenia: European consensus on definition and diagnosis: Report of the European Working Group on Sarcopenia in Older People. Age Ageing.

[37] Bohannon RW (1997). Comfortable and maximum walking speed of adults aged 20–79 years: Reference values and determinants. Age Ageing.

[38] Perera S, Mody SH, Woodman RC, Studenski SA (2006). Meaningful change and responsiveness in common physical performance measures in older adults. J Am Geriatr Soc.

[39] Fritz S, Lusardi M (2009). White paper: "Walking speed: The sixth vital sign". J. Geriatr. Phys. Ther..

[40] Choudhury R (2020). Viime: Visualization and integration of metabolomics experiments. J. Open Source Softw..

[41] Lakens D (2013). Calculating and reporting effect sizes to facilitate cumulative science: A practical primer for t-tests and ANOVAs. Front. Psychol..

[42] Brydges CR (2019). Effect size guidelines, sample size calculations, and statistical power in gerontology. Innov. Aging.

[43] Saville DJ (1990). Multiple comparison procedures: The practical solution. Am. Stat..

[44] Bailar JC, Mosteller F (1988). Guidelines for statistical reporting in articles for medical journals. Amplifications and explanations. Ann. Intern Med..

